# Laparoscopic dismembered pyeloplasty combined with port entrance flexible renoscopic lithotripsy

**DOI:** 10.1590/S1677-5538.IBJU.2017.0401

**Published:** 2018

**Authors:** Kaan Gokcen, Gokhan Gokce, Gokce Dundar, Resul Cicek, Halil Gulbahar, Emin Yener Gultekin

**Affiliations:** 1 Department of Urology, Cumhuriyet University Faculty of Medine, Sivas, Turkey; 2 Department of Urology, Cizre State Hospital, Cizre, Turkey

## Abstract

**Introduction:**

Ureteropelvic junction obstruction and concomitant calculus disease may coexist. We demonstrate our use of flexible renoscopy during laparoscopic pyeloplasty for caliceal stone removal.

**Patient and methods:**

A 28-year-old female patient presented with recurrent attacks of flank pain of two years duration. When noncontrast-CT and DTPA were performed, the patient was diagnosed with ureteropelvic junction stenosis and 3 stones with a total burden of 14mm in the lower pole of right kidney. After pneumoperitoneum was established in right flank position, three 10mm trocars were placed including one camera port. 5mm trocar was placed for convenience to retraction and dissection. The surgery was uneventful, with no operative complications or evidence of intra-abdominal bleeding.

**Results:**

The duration of the surgery was 110 minutes. The amount of bleeding was 30ml. On the postoperative 2nd day, the urethral catheter was removed and the patient was discharged on the fourth day postoperatively. Stent removal was done on the 3rd postoperative week and retrograde pyelogram showed normal ureter. Post-operative follow-up with ultrasound showed that hydronephrosis had regressed.

**Conclusions:**

Laparoscopic pyeloplasty and concomitant flexible renoscopy through lowermost trocar with basket extraction is a simple, attractive alternative for the simultaneous treatment of ureteropelvic junction obstruction presenting with coexisting nephrolithiasis. This method is useful and feasible, with minimal invasiveness and an early post-operative recovery.

## ARTICLE INFO

**Available at: http://www.intbrazjurol.com.br/video-section/20170401_Gokcen_et_al**

**Int Braz J Urol. 2018; 44 (Video #15): 1049-1049**

